# Electrically and all-optically switchable nonlocal nonlinear metasurfaces

**DOI:** 10.1126/sciadv.adh2353

**Published:** 2023-08-16

**Authors:** Mukesh Sharma, Mai Tal, Cormac McDonnell, Tal Ellenbogen

**Affiliations:** ^1^ Department of Physical Electronics, Faculty of Engineering, Tel-Aviv University, Tel-Aviv 6779801, Israel.; ^2^ Center for Light-Matter Interaction, Tel-Aviv University, Tel-Aviv 6779801, Israel.

## Abstract

Nonlocal effects on metasurfaces play an important role to achieve high-*Q* spectral selectivity, beneficial for development of multifunctional, multispectral integrated optics. In addition, they enhance the optical interaction and promote a variety of nonlinear effects, including frequency conversion and stimulated scattering. Active tuning of nonlocal nonlinearity is highly desirable for sensing and signal processing but was hardly explored until now. Here, we show drastic electric and all-optical tunability of nonlocal second-harmonic generation (SHG) from nonlinear metasurface, functionalized with a twisted nematic liquid-crystal (LC) layer. The addition of LC results in the emergence of strong nonlocal SHG, due to a surface lattice resonance of the system. We demonstrate a notable enhancement of SHG on resonance, more than 25 dB electrical switching amplitude, and all-optically induced phase transition imprinted on SHG. Our results on dynamic nonlocal effects introduce a very promising route for active nonlinear optical metadevices at the nanoscale.

## Teaser

Active switching of nonlocal nonlinear metasurfaces based on hybrid liquid-crystal-metasurface composite system is demonstrated.

## INTRODUCTION

Rapid recent developments in the field of nonlinear metasurfaces make them a highly promising platform to obtain a variety of nonlinear optical effects at the nanoscale (*1*–*5*). These effects include frequency conversion, harmonic generation, wave mixing, and ultrafast electro-optical and all-optical modulation (*6*–*14*). The manifestation of these effects in metasurfaces offers additional degrees of freedom to realize novel nonlinear metadevices with a wide range of potential applications such as nanoscale coherent light sources, ultrafast optical switching, sensing and modulation, nonlinear holography, super-resolution imaging, terahertz generation, topological photonics, generation of entangled photon pairs, quantum computing, and information processing (*15*–*24*).

To promote the nonlinear nanoscale effects on the metasurfaces, it is important to enhance the interaction with light and strongly confine it to the metasurface building blocks. This confinement can be achieved by exciting localized surface plasmon resonances (LSPRs) on metallic inclusions (*25*–*27*) or Mie resonances on dielectric inclusions (*28*, *29*). In addition, nonlocal metasurface modes, such as surface lattice resonances (SLRs) can be used. These modes emerge when a hybrid photonic-plasmonic coupling occurs between LSPR and the diffractive orders of the meta-atom array at the Rayleigh anomaly (RA) condition. Because nonlocal SLR modes exhibit high-*Q* resonance with narrow spectral linewidth, these modes can also drastically enhance the light-matter interaction (*30*–*33*) leading to pronounced nonlinear effects (*34*–*42*).

Recently, it was shown that the SLRs can be used to strongly enhance and engineer various nonlinear processes on metasurfaces such as second-harmonic generation (SHG), third-harmonic generation and higher harmonic generations (*34*–*38*). Yet, these improved nonlinear metasurfaces are still static in nature, which limits their functionality. Therefore, it is important to explore ways to achieve tunable and switchable nonlocal nonlinear effects that can be used for the development of next-generation active multifunctional nonlinear metasurfaces at the nanoscale. For this goal, liquid crystals (LCs) are highly promising active materials that can be integrated with the metasurfaces and efficiently controlled by external stimuli such as electric or magnetic fields, light, and temperature (*43*). In recent years, LC-based reconfigurable metasurfaces have attracted great deal of attention, due to a variety of demonstration of efficient active control of their linear optical response (*44*–*52*). It was also shown that the nearfield orientation of the LCs affects the local nonlinear optical response (*53*, *54*), opening the door to previously unknown ways to control the optical nonlinearity of metasurfaces.

Here, we use an integrated LC-nonlinear metasurface platform to show drastic tunability of nonlocal SHG both electrically and all-optically. Specifically, we use the polarization-rotating property of a twisted nematic LC (TNLC) layer, hybridized with nonlinear plasmonic metasurfaces. We observe that the SH signal is highly sensitive to polarization-dependent SLR that emerges because of the introduction of the LC layer. This SLR shows notable enhancement of the nonlocal SHG. By electrically aligning the TNLC layer, we modulate this nonlocal SHG with a modulation amplitude of more than 25 dB. In addition, we show that the excitation of the sample at the SLR wavelength above a certain threshold power can optically induce a nematic-to-isotropic (N-I) phase transition of the LC followed by an abrupt increase of the nonlocal SH signal.

## RESULTS

### Working principle of active tuning mechanism of the nonlocal effects

**Fig. 1.**
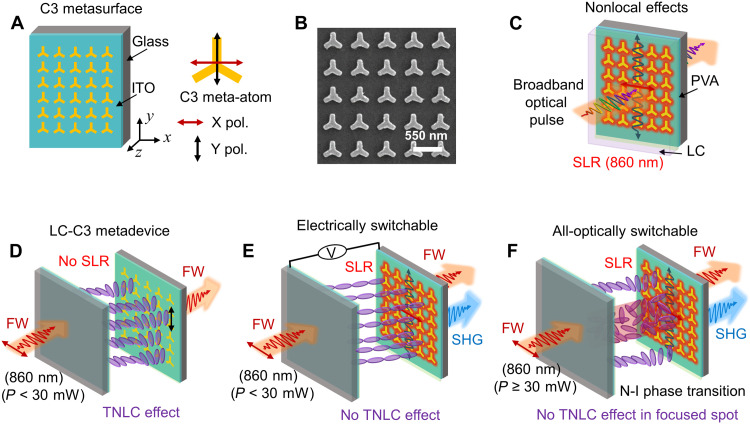
Illustration of nonlocal effects in liquid crystal (LC)–threefold rotational symmetry (C3) metadevice. (**A**) Schematic of metasurface with C3 symmetry meta-atoms fabricated on indium tin oxide (ITO)–coated glass substrate. (**B**) Scanning electron micrograph of the fabricated metasurface before spin coating of the PVA layer. Scale bar, 550 nm. (**C**) Schematic view of excitation of *x*-polarized dependent nonlocal surface lattice resonance (SLR) mode (860 nm). (**D**) Schematic representation of the working principle of the twisted nematic LC (TNLC) layer. The TNLC layer rotates the polarization of incident *x*-polarized light by 90° and converts it to y-polarized light. Schematic representation of (**E**) electrical switching and (**F**) all-optical switching of second-harmonic generation (SHG) at SLR. The metadevice is excited at an SLR wavelength of 860 nm that generates SH signal when *x*-polarized light interacts with the metasurface. The N-I phase transition is observed at *P* = 30 mW. FW, fundamental wavelength.

Figure 1 illustrates the general concept of nonlocal effects and their active tuning mechanism. Schematic and scanning electron micrograph of the fabricated metasurface are shown in Fig. 1 (A and B, respectively). The metasurface is composed of 30 nm thick gold meta-atoms with threefold rotational symmetry (C3), arranged in a square lattice of a 550 nm period. The metasurface is fabricated on indium tin oxide (ITO)–coated glass substrate and subsequently encapsulated in a thin (≈6 μm) TNLC cell, called LC-C3 metadevice (see Fig. 1, C and D). The details of the fabrication and assembly of the LC-C3 metadevice are given in Materials and Methods. The lattice parameters of the LC-C3 metadevice support a strong nonlocal SLR mode at a fundamental wavelength (FW) of 860 nm, only when *x*-polarized incident light interacts with the metasurface (Fig. 1C). The polarization-selective SLR originates in the LC-C3 metadevice due to the symmetry mismatch of the C3 meta-atoms arranged in the square lattice. This SLR wavelength for excitation at normal incidence can be estimated by λ_SLR_ = *p* × *n*_eff_, where, *p* and *n*_eff_ are the lattice period and effective refractive index of the surrounding environment including PVA and LCs, respectively. By using the parameters *p* = 550 nm and *n*_eff_ ≈ 1.565, we obtain λ_SLR_ ≈ 860 nm. The excitation of the SLR (red color) is accompanied by strong SHG (blue color) (see Fig. 1, E and F). The polarization-dependent nonlocal SLR and SHG in the C3 metasurface can be actively controlled by the LC matrix. This control can be achieved either electrically (see Fig. 1E) or all-optically (see Fig. 1F). Specifically, the all-optical control mechanism is based on nonlinear thermo-optic polarization switching in a TNLC layer, controlled by laser-induced phase transition in the NLCs (*46*). The origin of the N-I phase transition is achieved by local heating of the TNLC layer by using short femtosecond laser pulses specifically at the SLR wavelength of 860 nm. Beyond a threshold input power, the local temperature becomes higher than the critical temperature required for N-I phase transition [for details, please see our previous published results in (*46*)]. The threshold input power for all-optically induced N-I phase transition is observed at *P* = 30 mW. Below this threshold power (*P* < 30 mW), the LC-C3 nonlocal nonlinear effects can be electrically controlled. When the input power is sharply equal or greater than 30 mW (*P* ≥ 30 mW), the same effects can be all-optically controlled. As we show next, by exploiting these interesting electrical and all-optical switching mechanisms, it is possible to realize widely tunable nonlinear metasurfaces.

### Linear characterization of the hybrid LC-C3 metadevice

**Fig. 2.**
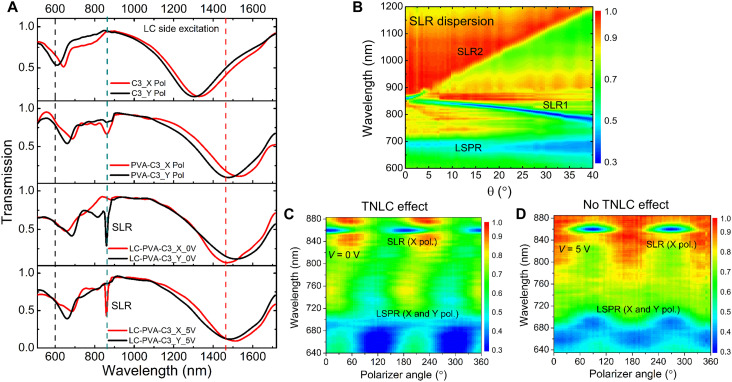
Linear characteristics of the hybrid liquid crystal (LC)–threefold rotational symmetry (C3) metadevice. (**A**) Transmission spectra of the bare C3 metasurface, PVA-C3 sample, and LC-PVA-C3 sample for *x*- and *y*-polarized incident light, respectively. A strong nonlocal surface lattice resonance (SLR) mode at 860 nm wavelength is observed when *x*-polarized incident light interacts with the metasurface in LC-PVA-C3 sample. The effect of twisted nematic LC (TNLC) layer with applied voltage is clearly observed. No SLR mode is observed in the bare C3 metasurface. However, a very weak SLR mode is observed in PVA-C3 sample. (**B**) Angle-dependent SLR dispersion curves for LC-PVA-C3 sample in the nematic phase, where θ is the angle in the *yz* plane. Polarization-dependent excitation of SLR mode with applied voltage (**C**) *V* = 0 V (TNLC effect) and (**D**) *V* = 5 V (no TNLC effect), respectively. LSPR, localized surface plasmon resonances.

Figure 2A shows, from top to bottom, the measured linear transmission spectra of the bare metasurface, after PVA coating (PVA-C3 sample), after integration with LC layer (LC-PVA-C3 sample) for *x*- and *y*-polarized light at normal incidence. The details of the experimental setup for the linear characterization are given in Materials and Methods. Coating the sample with the LC layer results in the emergence of a sharp dip in the transmission spectrum when *x*-polarized light interacts with the metasurface. This dip indicates the excitation of an SLR mode that is caused by a strong optical coupling between the individual LSPR of the C3 meta-atoms and the in-plane photonic mode of the lattice at the RA condition. To confirm the SLR excitation, the angle-dependent dispersion is measured for the case of z-oriented LC director (5 V) and presented in Fig. 2B. The LSPR and SLR can be clearly identified in the measurements. A comparative variation of polarization-dependent SLR excitation at θ = 0° and θ = 4° with applied voltage 5 V, and voltage-dependent SLR excitation at θ = 4° when the sample was excited with both *x-* and *y-*polarizations, is shown in fig. S1 (A and B, respectively), where θ is the angle of rotation of sample in the *yz* plane. In addition, we also measured the polarization-dependent excitation behavior of the system by varying the input polarizer angle between 0 and 360 degrees at an applied voltage *V* = 0 V (TNLC effect) and *V* = 5 V (no TNLC effect), as shown in Fig. 2 (C and D, respectively). It can be seen that the SLRs are excited only for *x*-polarized light and that it can be switched through electrically controlling the TNLC layer.

### Excitation and electrically switchable nonlocal SHG at SLR

**Fig. 3.**
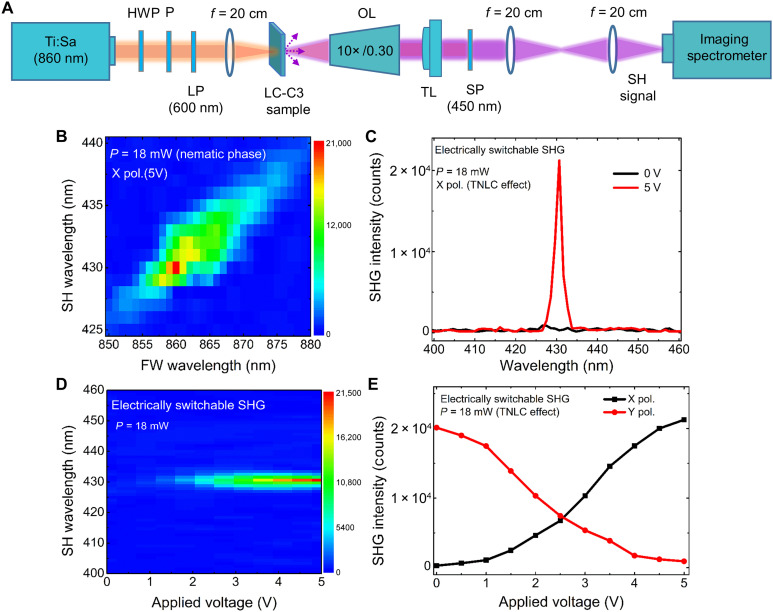
Excitation and electrical tuning of nonlocal SH signal. (**A**) Schematic of the experimental setup that consist of a Ti:Sa light source, half-wave plate (HWP), polarizer (P), long-pass filter (LP), three focusing lenses (*f* = 20 cm), liquid crystal (LC)–threefold rotational symmetry (C3) metadevice, objective lens (OL), tube lens (TL), short-pass filter (SP), and the imaging spectrometer. (**B**) Measurement of second-harmonic generation (SHG) excited at normal incidence for *x*-polarized fundamental wavelength (FW) with wavelength varying between 850 and 880 nm. The voltage on the cell is 5 V orienting the LCs in the *z* direction. It is clearly observed that the SH signal is strongly enhanced with narrow linewidth at the surface lattice resonance (SLR) wavelength (860 nm). (**C**) Electrical switching of the SHG at 0 and 5 V. (**D**) 2D plot for variation in SHG intensity with applied voltage 0 to 5 V. (**E**) Variation in SH signal with applied voltage when *x*- and *y*-polarized light excites. TNLC, twisted nematic LC.

A schematic of the experimental setup for the measurement of the SH signal from the LC-C3 metadevice and their active switching is shown in Fig. 3A (see also Materials and Methods). The linearly polarized input beam at FW (≈860 nm, orange color) from a Ti:Sa pulsed laser source is passed via half-wave plate, polarizer, and a 600 nm long-pass (LP) filter and weakly focused on the device with a lens (*f* = 20 cm). The SH signal (purple color) generated from the device is collected by an infinity-corrected microscope objective, followed by a tube lens, and the FW is filtered by a 450 nm short-pass (SP) filter. Then, the SH signal is captured and measured by an imaging spectrometer. To check the spectral dependence of the SH signal of the system, we first put a voltage of 5 V that aligns the LC in the *z* direction. Then we excite the metasurface with *x*-polarized FW and vary the FW wavelength between 850 and 880 nm. Figure 3B shows the SH signal versus FW and SH wavelength for a pump power of 18 mW. The diagonal signal confirms the measurement of the SH. The corresponding line plot of the peak SH signal versus the FW wavelength, shown in fig. S2 in the Supplementary Materials, reveals an SH enhancement due to the SLR by a factor of ≈10^4^ compared to no SLR case. Snapshots of demonstration of SHG at the chip level with and without FW are shown in fig. S3 (A and B, respectively). Note that the enhanced SH signal is observed only when *x*-polarized light interacted with C3 meta-atoms because of the excitation of the SLR mode.

The influence of the external electric field on the SHG signal at low power *P* = 18 mW is further studied. By switching the applied voltage from 0 to 5 V (or vice versa), we can completely turn on (or shut off) the SH signal with a large extinction ratio of >25 dB, as shown in Fig. 3C. Figure 3D shows the two-dimensional (2D) plot for the SH signal versus applied voltage from 0 to 5 V. By increasing applied voltages from 0 to 5 V, the SH signal increases gradually. The effect of applied voltages on polarization-dependent SH signal excited by *x*- and *y*-polarized incident light is shown in Fig. 3E (see also fig. S4, A and B). This shows the ability for both on-off switching and continuous electro-optics modulation of the nonlocal SHG.

### All-optically switchable nonlocal SHG at SLR

**Fig. 4.**
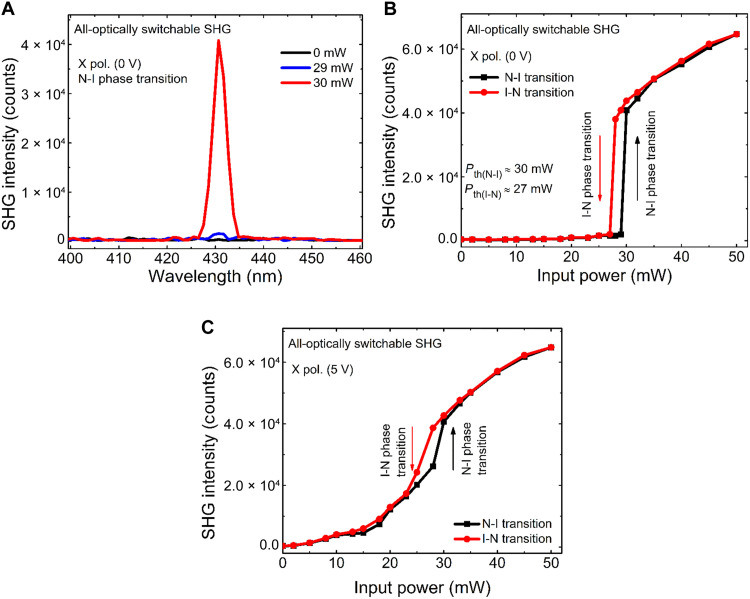
All-optically switchable SH signal. (**A**) Measured second-harmonic (SH) signal for *P* = 29 mW and *P* = 30 mW, below and above threshold, respectively. (**B**) SH signal versus input power at 0 V for *x*-polarized incident light. At threshold power *P* = 30 mW, SH signal is abruptly enhanced because of optically induced phase transition. (**C**) SH signal versus input power at 5 V for *x*-polarized incident light. A slight step-like increment is observed near 30 mW at nematic-to-isotropic (N-I) phase transition. SHG, second-harmonic generation; I-N, isotropic-to-nematic.

The SH signal can also be controlled by an all-optically induced N-I phase transition. To study this effect, we measure the SH dependence on pump power. As shown in Fig. 4A for the case of *x*-polarized excitation and without applying an electrical voltage, the SH signal is almost negligible for pump power below ~30 mW. At *P* = 30 mW, an abrupt enhancement of SH signal appears because of transition to isotropic phase and vanishing the TNLC configuration. This results in excitation of the metasurface layer with *x*-polarized light, which excites the SLR and SH signal, simultaneously (Fig. 4, A and B). The corresponding line plots are shown in fig. S4 (C and D) in the Supplementary Materials. A hysteresis behavior is also observed during isotropic-to-nematic (I-N) phase transition with slightly lower threshold power (*P* ≈ 27 mW) (Fig. 4B).

Figure 4C shows the enhancement in SH signal with increasing input power when applied voltage is fixed at 5 V and the LC molecules are oriented in the *z* direction. A nearly quadratic dependence on the input power before phase transition (*P* < 30 mW) is obtained because of the second-order nonlinear optical process. A step-like increment in SH signal appears exactly at the phase transition (*P* = 30 mW), followed by a sublinear increase for higher excitation power. This may be due to modification of the thermal properties of LCs. A similar hysteresis behaviour is observed during I-N phase transition (see Fig. 4C). From our active switching results, it is evident that the electrical switching mechanism dominates when *P* < 30 mW due to the existence of the nematic phase and all-optical switching dominates when *P* ≥ 30 mW due to the optically induced N-I phase transition.

## DISCUSSION

We experimentally demonstrate a strong active tuning of nonlocal nonlinear effects, both electrically and all-optically, in a hybrid nonlinear LC-C3 metadevice. The nonlocality in the system is supported by a polarization-dependent SLR that emerges with the introduction of the LC molecules to the device. We find that the SH signal due to the SLR is drastically enhanced. We also demonstrate that the system exhibits an optical excitation power threshold for N-I phase transition, with abrupt modification in the SH signal. This dynamic transition origins from large laser induced thermo-optic nonlinearities of the system (*46*). Below the threshold, the SH signal can be switched on and off or modulated electrically. Around the threshold power, the SH signal can be switched on and off all-optically. This demonstrates that merging nonlinear metasurface and LC active materials opens the door to construct new types of electrically and optically controlled active nonlinear devices. These devices may find applications in optical signal processing, on-chip communications, and sensing.

## MATERIALS AND METHODS

### Fabrication of integrated LC-C3 metadevice

The nonlinear C3 metasurface was fabricated by using standard electron-beam lithography technique. The gold C3 meta-atoms are arranged in a square lattice of a 550 nm period with a thickness of 30 nm and fabricated on ITO-coated glass substrate (≈30-nm-thick ITO layer). This substrate with the C3 meta-atoms was spin coated with an alignment layer of PVA (≈200-nm thickness). Another ITO-coated glass substrate was spin coated with the same alignment layer of PVA (≈200-nm thickness). Both PVA-coated substrates were baked at 120°C for 30 min and subsequently cooled to room temperature. The baked PVA layers were then rubbed mechanically in the orthogonal directions to each other to promote a TNLC configuration of LC molecules. A 6-μm-thick Mylar spacer layer (Sigma-Aldrich) was used between two substrates, and then, it was assembled together by using ultraviolet (UV)–curable adhesive NOA61 followed by UV curing. After this stage, the electrical contacts were made on the top and bottom substrates using a thin aluminum wire and silver conducting paint. The nematic 5CB LC was infiltrated into the empty hybrid LC-C3 metadevice by capillary action. During the LC infiltration procedure, the fabricated device was kept on a hot plate that maintained a temperature of 50°C. At this temperature, the 5CB LC was in its isotropic phase, which had lower viscosity than its nematic phase, and therefore, it could be easily infiltrated into the gap. The filled device was cooled down to room temperature for approximately 2 hours, and then, the two open sides were sealed using the same UV-curable adhesive.

### Experimental setup and excitation of SLR and SH signal

The linear and nonlinear optical response of the LC-C3 metadevice was characterized by using linearly polarized incident light. For linear characterization, a broadband supercontinuum white-light source (SuperK COMPACT, NKT Photonics) with emission wavelengths between 450 and 2400 nm was used. The visible (VIS) and near-infrared (NIR) transmission spectra were measured by two spectrometers Avantes (AvaSpec-3648) and Ocean optics (NIRQuest-512), respectively. The obtained data were combined and plotted in a single plot showing the full VIS-NIR transmission spectrum. Here, we mention that, in all measurements, the transmission spectrum where normalized with respect to the clean area in the respective samples, with no metasurface, under the same excitation conditions. All experiments were performed to study the SLR and SHG excitation when incident light was impinging from the LC side. For the measurement of SLR dispersions at varying oblique angles of incidence, the samples were placed on a motorized *xyz*-rotational stage (Thorlabs). For nonlinear characterization, a femtosecond Ti-sapphire laser (Chameleon OPO VIS, pulse width of ∼140 fs, repetition rate of 80 MHz), was used as the FW source operated at an SLR wavelength of 860 nm. The FW was filtered spectrally by using LP filter (≈600 nm) to avoid residual SH from the optical parametric oscillator (OPO), and its power and polarization were controlled by a half-wave plate and a polarizer (Fig. 3A). Emission from the sample (i.e., SH signal) was collected by an infinity-corrected objective lens (Nikon, 10×/0.30) and filtered spectrally. After filtering the fundamental light using an SP filter (450 nm), the SHG signal was spectrally analyzed with an Andor imaging spectrometer based on an electron-multiplying charge-coupled device detector (Andor Shamrock 303i). For electrical switching, the FW (860 nm) power was 18 mW, and the device was connected to a signal/function generator (Stanford Research Systems DS345, 30 MHz) to provide an ac square signal wave of frequency of 1 kHz to modulate the optical properties of the LC molecules.
