# Supplementary material for: Electrically and all-optically switchable nonlocal nonlinear metasurfaces
Source: Sci Adv. 2023 Aug 16;9(33):eadh2353. doi: 10.1126/sciadv.adh2353 (PMC10431712; doi:10.1126/sciadv.adh2353)
Supplement: Supplementary file 1 — Figs. S1 to S4 [file sciadv.adh2353_sm.pdf]

Science Advances
AAAS

Supplementary Materials for

Electrically and all-optically switchable nonlocal nonlinear metasurfaces

Mukesh Sharma *et al.*

Corresponding author: Mukesh Sharma, mukeshsharma@mail.tau.ac.il; Tal Ellenbogen, tellenbogen@tauex.tau.ac.il

Sci. Adv. **9**, eadh2353 (2023)
DOI: 10.1126/sciadv.adh2353

This PDF file includes:

Figs. S1 to S4

Fig. S1.

Figure S1 shows the comparative variation of polarization-dependent SLR excitation at $\theta = 0°$ and $\theta = 4°$ with applied voltage 5V, and voltage-dependent SLR excitation at $\theta = 4^o$ when the sample was excited with both *x*- and *y*- polarizations. From Fig. S1A, it can be observed that *x*-polarized light excites a very strong SLR at $\theta = 0°$ whereas *y*-polarized light excites a very weak or almost negligible SLR at $\theta = 0°$. At $\theta = 4°$, the *x*-polarized light-dependent strong SLR is split into two SLRs with equal magnitudes, while *y*-polarized light still does not excite a significant SLR (see Figs. S1A and S1B). The voltage-dependent measurement (at $\theta = 4°$), as shown in Fig. S1B, also confirm the effect of strong polarization-dependent SLR excitation and SLR mode splitting with applied voltages.

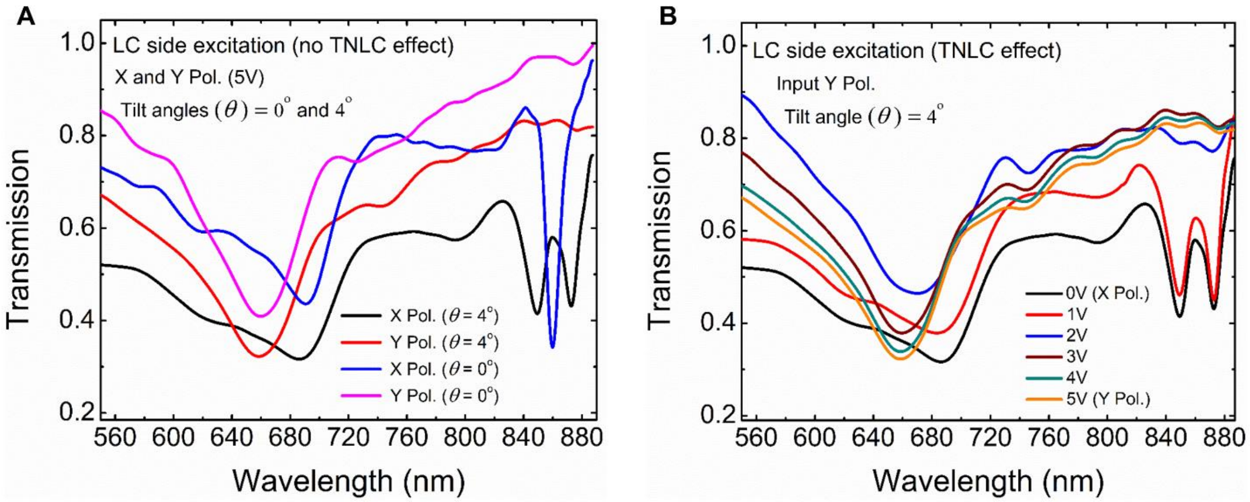

Fig. S1. Demonstration of polarization and voltage-dependent SLR. Comparative variation of (**A**) polarization-dependent SLR excitation at $\theta = 0^o$ and $\theta = 4^o$ with applied voltage 5V, and (**B**) voltage-dependent SLR excitation at $\theta = 4^o$ when the sample was excited with both *x*- and *y*-polarizations, respectively.

Fig. S2.

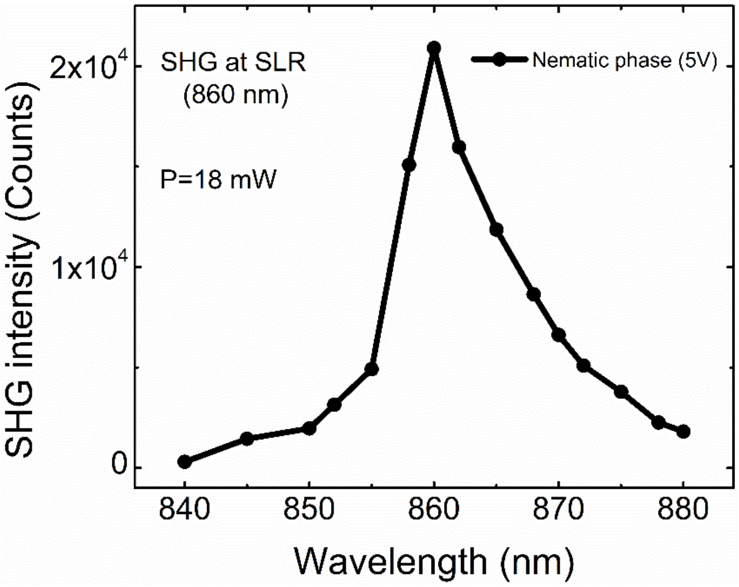

Fig. S2. Excitation of nonlinear SHG at SLR. Measurement of SHG for different FWs near the SLR wavelength, at normal incidence for *x*-polarized incident light for P=18 mW and V= 5V (nematic phase). It is clearly observed that the SH signal is strongly enhanced with narrow linewidth at the SLR wavelength (860 nm).

Fig. S3.

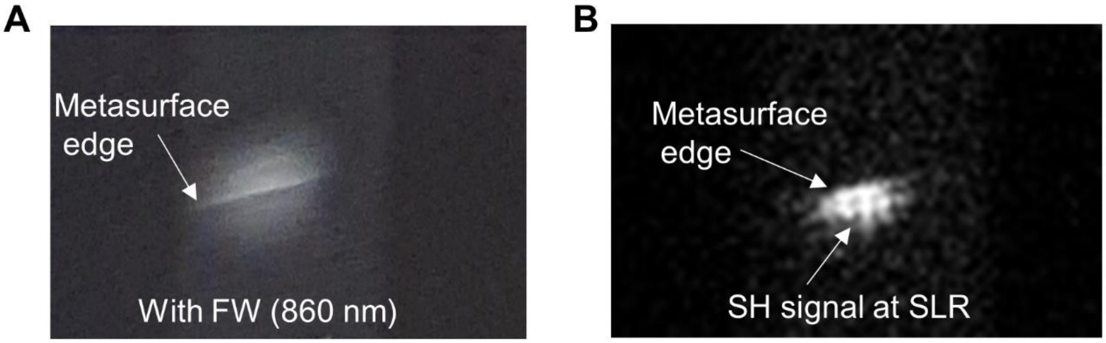

Fig. S3. Demonstration of SHG at SLR. (**A**) Snapshot of C3 metasurface edge for *x*-polarized incident light with input power P = 18 mW and V=5V at FW 860 nm. The SLR propagation into the substrate at 860 nm wavelength can be clearly seen in figure. (**B**) Snapshot of C3 metasurface edge with generated SH signal for *x*-polarized incident light with input power P = 40 mW (isotropic phase) and V=0V without FW. A strong SH signal can be clearly seen at the edge of metasurface.

Fig. S4.

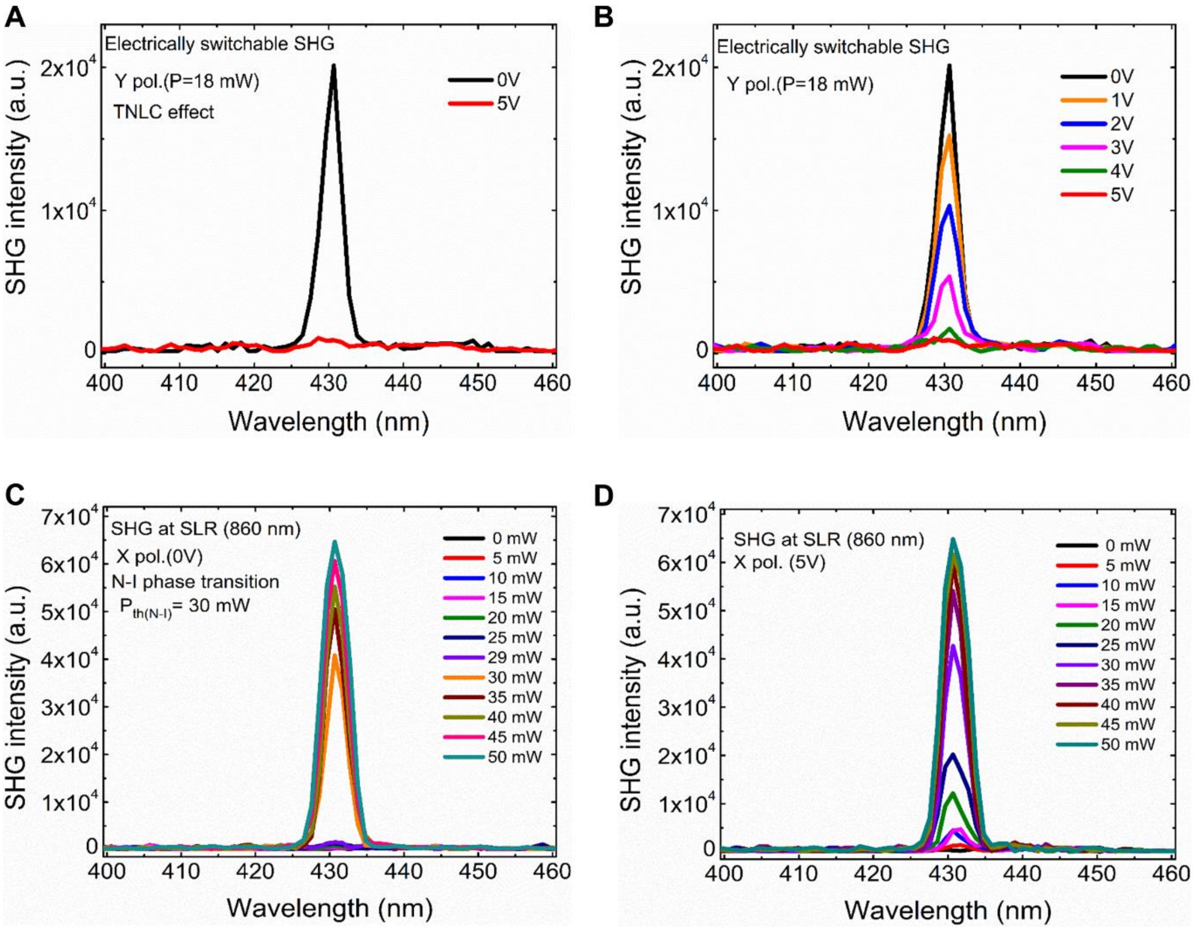

Fig. S4. Electrically and all-optically switchable SHG. Measured *y*-polarization-dependent SHG spectra with input power P=18 mW showing (**A**) electrical switching of SH signal at 0V and 5V, respectively and (**B**) variation in SHG intensity with applied voltage 0 to 5V. (**C**) Excitation and variation in SH signal with input power at 0V. At threshold power P =30 mW, SH signal abruptly enhanced with a factor of $\approx 10^4$. (**D**) Excitation and variation in SH signal with input power at 5V. A step-like enhancement is observed near 30 mW at N-I phase transition.
